# Rapid cardiac cine imaging using MACH

**DOI:** 10.1186/1532-429X-11-S1-P250

**Published:** 2009-01-28

**Authors:** Mark Doyle, Geetha Rayarao, Diane A Vido, Vikas K Rathi, Saundra Grant, June A Yamrozik, Ronald B Williams, Robert WW Biederman

**Affiliations:** 1 Allegheny General Hospital, The Gerald McGinnis Cardiovascular Institute, Pittsburgh, PA USA; 2 Allegheny General Hospital, Pittsburgh, PA USA

**Keywords:** Acceleration Factor, Ringing Artifact, SSFP Cine, Sparse Factor, Axis Acquisition

## Introduction

Previously, we developed a sparsely distribute k-space-time sampling approach termed BRISK, Block Regional Interpolation Scheme for K-space [1]. This approach allowed a nominal acceleration factor of 4 with good quality and low artifact. Others have developed alternative k-space-time sampling schemes, such as KT-BLAST and SLAM [2, 3]. We note that even at high acceleration factors, KT-BLAST/SLAM allowed a smooth transition from frame to frame, while BRISK experienced ringing artifacts. From these considerations we isolated key features that contribute to a successful k-space-time sparse sampling scheme:

1) Update of k-space should be rapid near the center and lower near the periphery (as in BRISK) to capture highly dynamic features.

2) Update of k-space should smoothly vary over time (as in KT-BLAST/SLAM) avoiding sudden transitions between k-space regions to result in smoother transition between frames.

**Figure 1:**
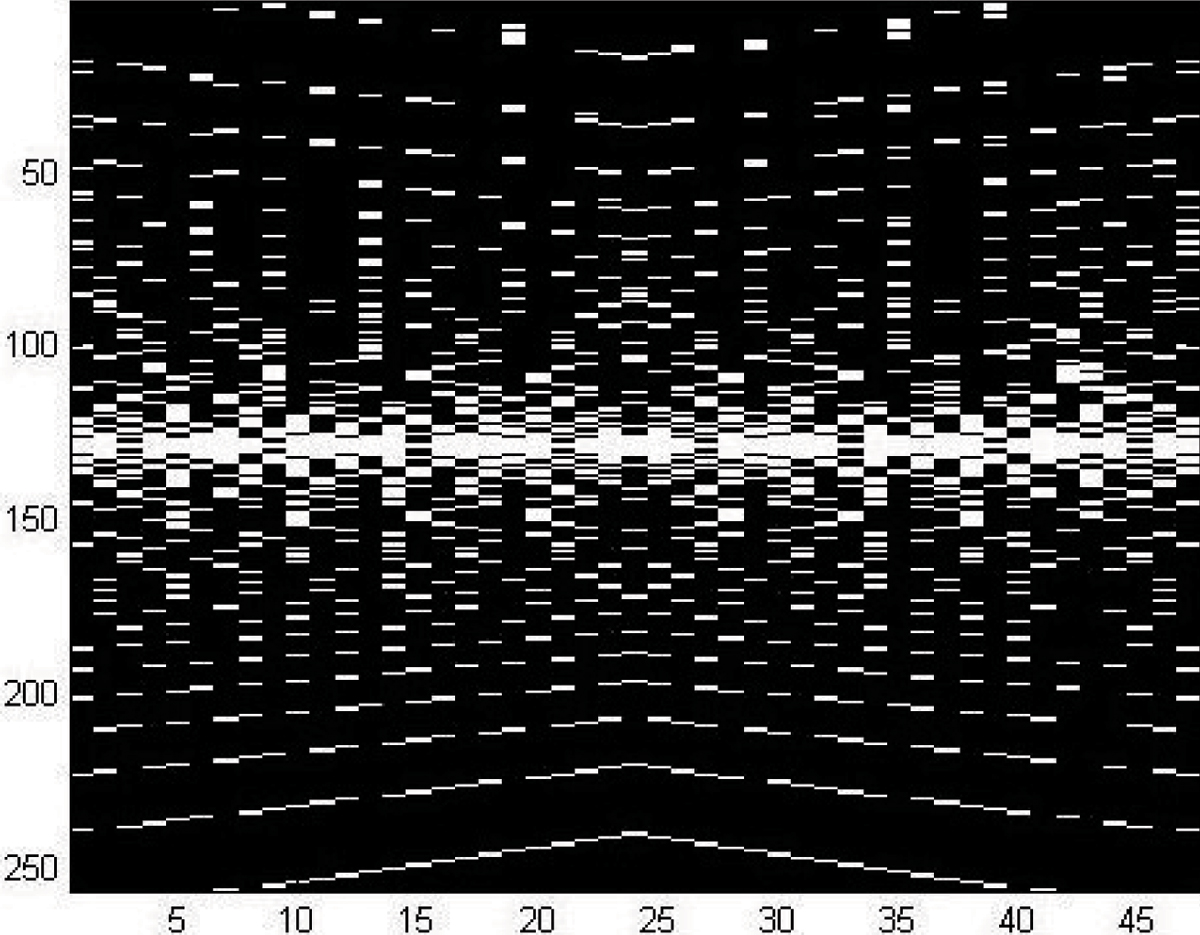
Figure 1

From these design principles we developed a new sparse k-space-time sampling scheme, MACH, Multiple Acceleration Cycle Hierarchy. MACH incorporates a gradually changing rate over time, starting with the highest rate near the center of k-space and becoming progressively sparser towards the periphery, Figure 1 shows the k-space-time sampling scheme. In MACH, the progressively decreasing sampling rates are not confined to integer steps, thereby providing greater opportunity for a smooth transition over the k-space-time domain. Data that are not directly sampled in MACH are interpolated prior to applying conventional Fourier transformation to generate the series of images. Since each frame incorporates a full set of k-space data, the SNR is similar to the conventional scan.

## Methods

Simulations were performed using fully acquired stead-state-free-precession (SSFP) cine image data to allow direct comparison of MACH and KT-BLAST/SLAM when using the same acceleration factors. Further, MACH was implemented on a 1.5 T scanner (GE, Milwaukee, WI). Using the SSFP cine acquisition, long and short axis acquisitions were acquired using the conventional examination and MACH applied with a net sparse factors ranging from 2 to 5, with matrices ranging from 224 to 336. Comparable cine acquisitions were acquired of the heart in 10 volunteers. The end-diastolic and end-systolic phases were identified and areas were planimetered and compared between the conventional and the MACH accelerated scans.

## Results

**Figure 2:**
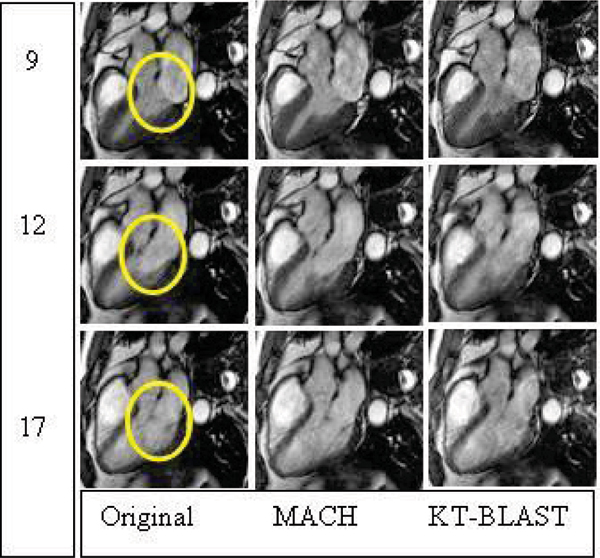
Figure 2

**Figure 3:**
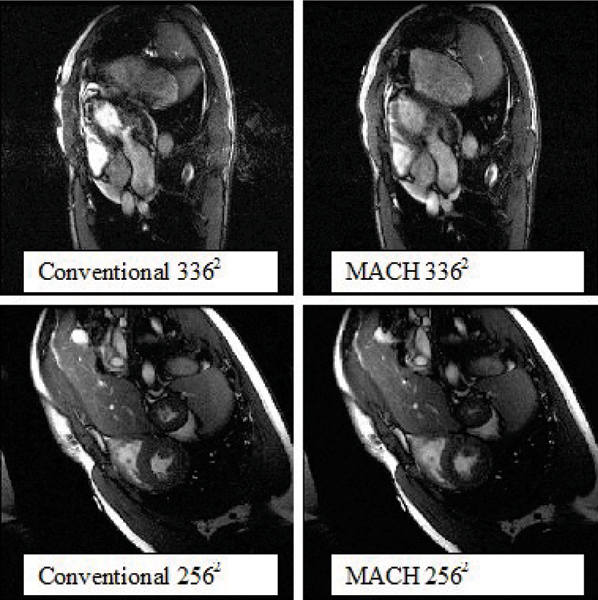
Figure 3

Simulations showed that for a moderate acceleration factor of 5, KT-BLAST/SLAM represented myocardial motion well, but lost detail of the relatively fast moving valvular features, while MACH still represented these features. In the *in vivo* acquisitions, the average MACH acceleration factor applied was 3.5 ± 1.1, end-diastolic and end-systolic ventricular chamber areas were not significantly different between the conventional and the accelerated MACH scans (p = 0.7, 0.6, respectively) and correlations were excellent at 0.99 for each. Compared to the conventional scan, there is no additional overhead with MACH. See Figures 2 and 3.

## Conclusion

In sparse k-space-time acquisition strategies, rapidly updating the central region of k-space is known to be important. We note that MACH achieves this condition very efficiently while also achieving a smooth transition of update rate between each region of k-space since MACH does not use a uniform or even a regular update rate. MACH was successfully implemented and shown to accurately represent cardiac regions with good fidelity.
